# Accessibility to Reproductive Technologies by Low-Income Beef Farmers in South Africa

**DOI:** 10.3389/fvets.2021.611182

**Published:** 2021-07-23

**Authors:** Nkhanedzeni Baldwin Nengovhela, Thinawanga Joseph Mugwabana, Khathutshelo Agree Nephawe, Tshimangadzo Lucky Nedambale

**Affiliations:** ^1^Department of Agriculture, Forestry, and Fisheries (South Africa), Pretoria, South Africa; ^2^Department of Agricultural Economics and Animal Production, School of Agricultural and Environmental Sciences, University of Limpopo, Polokwane, South Africa; ^3^Department of Animal Science, Tshwane University of Technology, Pretoria, South Africa

**Keywords:** body condition score, calving rate, assisted reproductive technologies, low-income beef sector, cattle stakeholders

## Abstract

This study address historical legacy of South Africa that has dual economies resembling low and high income beef sectors. Low-income herds are farmed mainly under communal village or land reform farms. The study focused on providing assisted reproductive technologies (ARTs) to the low-income sector including finding challenges to its implementation and adoption. The study was conducted in Limpopo, Mpumalanga and KwaZulu-Natal provinces using mixed methods that looked at cows and sectors stakeholders. Data collected and evaluated on cows included breed type, frame size, body condition, age parity, and lactation status. Cows were exposed to ART through synchronisation, oestrus detection, fixed time artificial insemination and pregnancy diagnosis. Qualitative data was collected to study perception of key stakeholders on ART implementation and adoption. Chi-Square Test was computed to determine the association among cow factors. Qualitative data was collected, coded and managed into themes using Nvivo Version 11. Themes that emerged were interpreted using critical social and systems thinking. Conception rate was not independent of provinces (*P* < 0.05), cow body condition score (BCS) and body frame size. KwaZulu-Natal cows had the highest conception rate at 66% (*P* < 0.05) than Limpopo (44%) and Mpumalanga (60%) provinces. Cows with a BCS higher than 3.5 had higher conception rate (*P* < 0.05) than those with BCS of <2.5 and 3. Interestingly, large framed cow size had higher conception rate than medium and small framed (*P* < 0.05) cows. The study achieved a 100% calf survival rate. Calving rate was influenced by body BCS, province and district (*P* < 0.05). Calving rate of 58% in Mpumalanga and 54% in KwaZulu-Natal was higher than that recorded in Limpopo at 36% (*P* < 0.05). Interestingly, cows with BCS of <2.5 had a higher calving rate than those with a higher body condition score of 3 (*P* < 0.05). Perception study results revealed many factors that could affect the adoption and implementation of ART in the study areas. The high success rate and above average reproductive performance led to North West and KwaZulu-Natal provinces adopting ART as part of their low-income beef sector support.

## Introduction

In South Africa, a dual system exists in terms of the livestock farming sector with a highly commercialised, high income and resourced sector at one end and the low-income to almost subsistence on the other end. The duality is extremely entrenched due to an extended biassed investment in agricultural infrastructure, technology, training, knowledge, and extension towards the highly commercialised sector. The low-income beef sector has a history of being neglected by value chain actors, research agencies, universities, public and non-governmental sectors even though donor organisations had always shown interest and the potential to leverage good livelihoods. The commercial livestock industry is highly sophisticated and implements appropriate technologies to ensure good value for the South African consumers but has been struggling to meet the demand of beef in the country. South Africa imported about 28.000 tonnes of beef in 2017 (1). The priority shifted to achieve the much-needed growth from the low-income sector is faced by persistent and wicked challenges requiring innovativeness to resolve. The low-income beef sector of South Africa, just like in many other Low and Middle-Income Countries (LMICs), still struggles to access inputs and services to drive productivity.

According to Statistics South Africa (2), the high-income herd holds about 60% of the national herd and the low-income herd sector commands a respectable 40%. In provinces like KwaZulu-Natal, the low-income beef herd holds a majority at 70% of the provincial herd. The common breeds of cattle that are found in the low-income beef sector are the Nguni, Bonsmara, Brahman, and other non-descript breeds. The low-income herd has little contribution to the formal national beef industry's income of R128 billion (2). Cattle productivity in communal lands is generally poor in terms of calving percentages (<40%), high calf mortality rates (>35%), low weaning weights (<180 kg at day 205), low post-weaning growth, and high mortality rate (3–5). These are some of the old problems besetting the sector and complemented by other socio-economic challenges (6). Under the low-income beef production systems, cows rarely conceive within a year of calving, calving intervals of 2–3 years are common (4, 5, 7). According to Stroebel et al. (8), the long calving interval could be attributed to the fact that few farmers wean calves and the majorities allow calves to run with the dams until natural separation occurs. The list of contributors to constraint productivity is vast and had been well-documented.

South Africa has over 483.270 farmers with beef herds of <10 animals, about 118.000 that own more than 10 animals, and only about 14.000 that owns more than 100 animals (2). Herd size was considered a major constraint to increasing cattle productivity and efficiency within the low-income herd in several studies (8–10). Attempts to address stocking rates, reproductive rates, growth rates, and access to markets are frustrated by the scale of production, and there were several attempts to address it through organisational rearrangements in most cases with little success. So, initiatives that aggregate these herds are a must to form bases for effective support and access to inputs and services.

Another constraint that affects the reproductive performance of this herd is the herd inventory that has females making up the largest proportion of the herd (8, 10). Farmers with small herds hope to grow their herds by keeping all females and use males for sale or as castrates for other uses. This leads to many households with no breeding bulls at the herd level or a very high bull to cows' ratio at the village level. The breeding is therefore left to chance at the communal grazing areas leading to inbreeding and reduced fitness (5, 6, 11). South Africa has a long history of performance testing and genetic evaluation, but none of these pricey sires can find their way into these herds other than through government or donor support, so superior animals are hardly used in the low-income herds.

Livestock farmers in the low-income sector in South Africa rely on extension services offered by the government, which is considered inadequate (5, 12). The challenges in the low-income beef sector are enormous, thus requiring some innovativeness. Any intervention strategies and actions will need to be built on a clear understanding of these challenges; otherwise, the status core will remain.

The commercial beef sector had relied on the use of technologies to support their productivity. Smallholder and low-income beef farmers can adopt and implement the same technologies to drive productivity in the sector. According to Ndove et al. (13) and Muzari et al. (14), key drivers of technology implementation and usage by rural farmers are assets, vulnerability, and institutions. The South African government, through its Technology Innovation Agency funding incubator, the Agricultural Research Council (ARC), three Provincial Departments of Agriculture, and two Universities implemented the Livestock Development Programme in 2012. The programme introduced assisted reproductive technologies (ARTs) in smallholder cattle farmers in Limpopo, Mpumalanga, and KwaZulu-Natal provinces. The programme was designed as an integrated initiative to address the problem of bull shortages, access to improved genetics, to improve reproductive performance of cows and barriers of adoption in the sector. ARTs seldom exist in communal and emerging farming systems in South Africa, not only because of the cost factor but also because the free support systems are not designed to implement them at the sector level. The project was to present the observable feasibility of implementing ARTs and improvement of reproductive performance under the low-income sector. The aim of this study was to evaluate the effect of the implementation of ARTs to improve production under the low-income beef sector to develop policy directives for successful implementation of the project among low-income farmers.

## Materials and Methods

### Study Area

**Figure 1:**
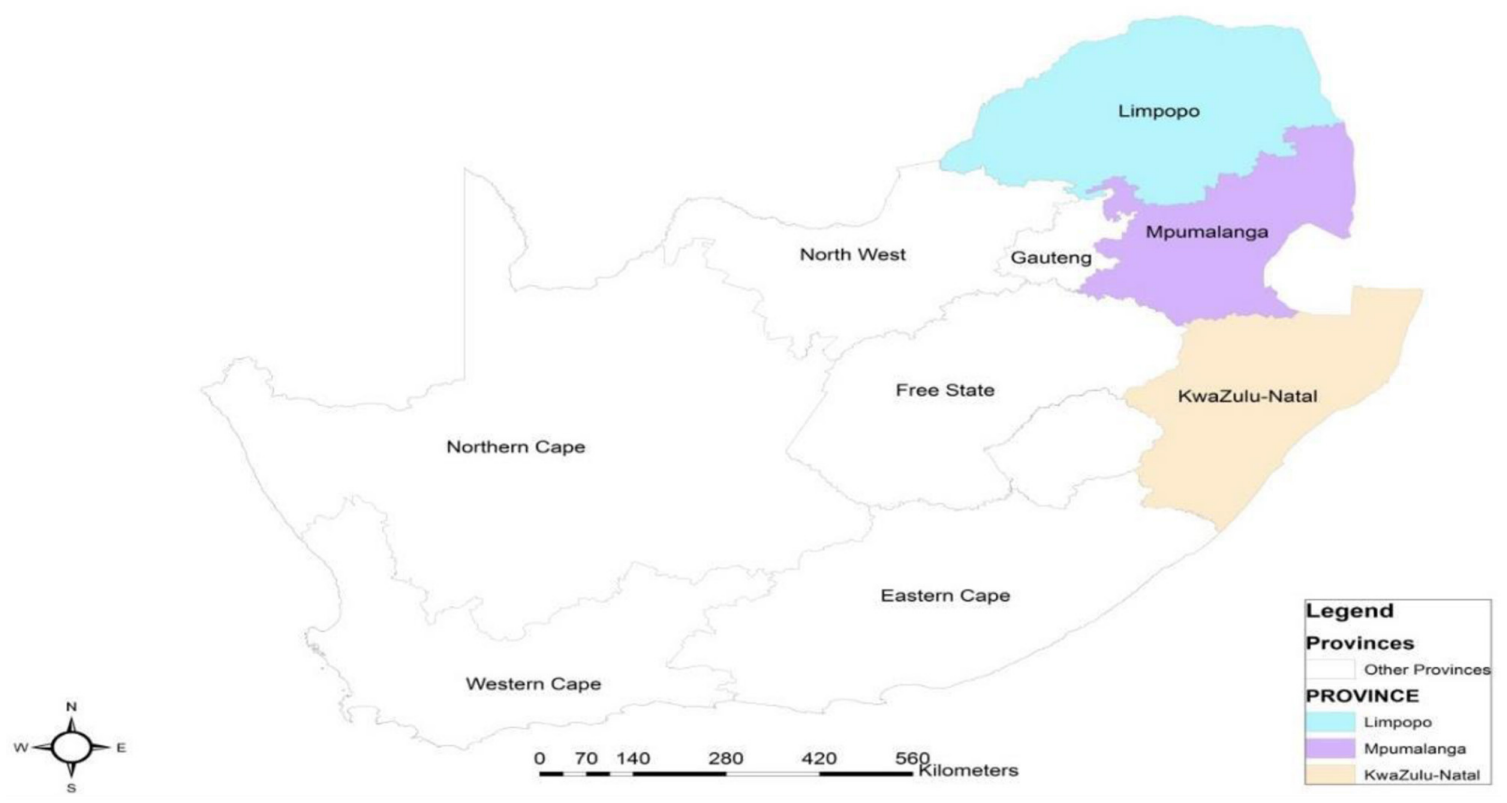
Map of the Republic of South Africa showing the study area.

The study used a multidisciplinary approach and was conducted in three provinces of South Africa, namely: Limpopo (LP), Mpumalanga (MP), and KwaZulu-Natal (KZN) provinces. The selected districts for the study were; Vhembe, Capricorn, Mopani and Waterberg (LP), and Gert Sibande and Ehlanzeni (MP), Zululand and Harry Gwala (KZN). A large part of these provinces is rural, with a large number of organised low-income beef cattle farmers. Limpopo province covers an area of 125.755 km^2^ and is located in the northern part of the country. Rainfall in the province ranges from 346 to 1,560 mm per annum. Its average summer and winter temperature are 27 and 15°C, respectively (15–17). Mpumalanga province covers a total area of 76.495 km^2^. The province is located in the north-eastern part of the country. Rainfall averages between 600 and 1,600 mm per annum with daily average summer and winter temperatures of 24 and 14.8°C, respectively (15, 18). KwaZulu-Natal is a province of 94.361 km^2^ in size. The province receives an average of 1,000 mm rainfall per annum. Summer temperatures average 28°C, and winter temperatures seldom fall below 17°C in mid-winter (15, 19). Figure 1 shows a map of the Republic of South Africa showing the study area.

### Identification of Key Stakeholder

A stakeholder analysis tool was used to determine stakeholder with power and interest in the implementation of the ART project (20). Key stakeholders who were identified and participated in the project were; Technology Innovation Agency (TIA), ARC, Provincial Departments of Agriculture, cattle farmers, and universities. TIA provided funding for the project; ARC was the main drivers of the project; Provincial Departments of Agriculture co-ordinated the project at a provincial level; cattle farmers willingly provided their cows to be used as experimental units; universities helped in providing study opportunities and funding for studies.

### Selection of Cows Used

Cows used in the trial were selected at random. Selected cows were then screened for age (4 years and older), non-pregnant, normal reproduction cycle, parity (should have given birth before regardless of the number), body condition score (≤2.5 to ≥3.5) and disease-free, especially contagious abortion. Cows were then grouped according to province, district, breed type, parity, age, frame size, lactation status, and body condition score ranging from 1 to 5 (21). The different breeds were identified by their phenotypic traits of resemblance to either the Nguni, Bonsmara, or Brahman type.

### Oestrous Synchronisation and Artificial Insemination

Experimental cows were synchronised using the ovsynch protocol that allows for fixed-time artificial insemination (FTAI) following synchronisation. On day 0, cows were given a dose of vitamin (Atlantic Gold®) to boost their immunity and body condition and were inserted in their vagina with controlled internal drug release (CIDR®, New Zealand) device containing 1.9 g progesterone. On day 8, CIDR® was removed, and cows were immediately inspected for pregnancy using both rectal palpation and ultrasound scanner. Those cows that were not pregnant were then immediately injected i.m with 2.5 ml of Estrumate (PGF2α) to stimulate ovulation. On day 9, cows were injected i.m with 1 ml of estradiol benzoate (EB) and then mounted with a heat mount detector (Kamar®, USA) on their tail head. The device change colour to red when a cow was mounted (indicating a positive response to the protocol).

Artificial insemination (AI) was performed 12 h after EB injection. Frozen-thawed semen of registered Nguni bulls of superior fertility was used. Semen quality was evaluated before insemination using Computer Aided Sperm Analysis (CASA) before insemination, and semen of high sperm motility (≥75) were used. Cows were inseminated twice at 12 h interval on day 10 and again on day 11 (late afternoon and early morning). Pregnancy diagnosis was performed 90 days later through both the use of a scanner and hand palpation.

### Statistical Analysis of Collected Data

The data collected were on province, district, breed type, parity, age, body condition score, frame size, and lactation status. The data collected was captured in Microsoft Excel 2013 and FREQ procedure of Statistical Analysis System (22) was used for descriptive statistics according to the collected data. Chi-Square Test of Independence were computed between dependent and independent variables. Qualitative data was collected using a semi-structured questionnaire that was developed and administered to identified stakeholder to determine their perception of the implementation and adoption of the project under the low-income beef sector. Qualitative data collected was analysed using Nvivo version 10 computer software package developed by QSR International Ltd. Whole sentences and paragraphs were coded, and emerging themes captured, analysed, and interpreted on how they could affect the implementation and adoption of ART under low-income beef sector using critical social theory and systems thinking (23).

## Results

### Oestrous Synchronisation Response and Conception Rate

The study recorded an oestrous synchronisation response and conception rate of 100 and 55%, respectively (Table 1). The computed Chi-Square Test of Independence showed that the conception rate was not independent of the province. Conception rates in MP (60%) and KZN (66%) were significantly higher (*P* < 0.05) than that obtained in LP. No significant difference (*P* > 0.05) was found between MP and KZN, and within districts of different provinces. However, Gert Sibande (62%) of the MP recorded the highest conception rate, whereas the least conception rate was recorded in the Mopani (31%) district of the LP. Chi-Square Test of Independence showed that breed type, and body frame size has no association with conception rate (Table 2). There was a small fraction of Afrikaner, Drakensberg, Simmentaler, and non-descript breeds that were classed as other, and were neglected in further discussions because of the small group's size. Brahman (63%) breed-type cows recorded the highest conception rate, whereas the least conception rate was recorded in Nguni (54%). Conception was independent of parity and age of the cows under communal and emerging farming systems (Table 3). Cows in fifth+ (71%) parity had the highest conception rate, whereas the least conception rate was recorded in second (51%) parity cows. Cows aged 8+ (83%) had the highest conception rate, whereas the least conception rate were recorded in cows aged 5 and 6 (both at 49%) years. The conception rate was not independent (*P* < 0.05) of body condition score (Table 4). Cows of body condition score of ≤2.5 and ≥3.5 had significantly higher (*P* < 0.05) conception rate than cows of body condition score of 3. There was no significant difference (*P* > 0.05) in the conception rate of cows of body condition score of ≤2.5 and ≥3.5. The lactation status of a cow was independent of conception (*P* > 0.05).

**Table 1:** Effect of province and districts on synchronisation response and conception rate of cows under communal and emerging farming systems.

**Variables**	**Number synchronised**	**Synchronisation response rate**	**Number of cows inseminated**	**Number conceived**	**Conception rate**	**Contribution towards conception**	**Chi-square value**	***P*-value**
**Province**								
Limpopo	108	100	108	47	43.52	17.22	10.6047	0.0050^*^
Mpumalanga	91	100	91	55	60.44	20.15		
KZN	83	100	74	49	66.22	17.94		
Total	282	100	273	151	55.31	55.31		
**Districts**								
Vhembe	48	100	48	25	52.08	9.16	3.5210	0.3180
Capricorn	37	100	37	13	35.14	4.76		
Mopani	13	100	13	04	30.77	1.47		
Waterberg	10	100	10	05	50.00	1.83		
Gert Sibande	69	100	69	43	62.32	15.75		
Ehlanzeni	22	100	22	12	54.55	4.39		
Zululand	55	100	46	30	65.22	10.99		
Harry Gwala	28	100	28	19	67.86	6.96		
Total	282	100	273	151	55.31	55.31		

* *Significant relationship (not independent) (P < 0.05)*.

**Table 2:** Effect of breed and body frame size on synchronisation response and conception rate of cows under communal and emerging farming systems.

**Variables**	**Number synchronised**	**Synchronisation response rate**	**Number of cows inseminated**	**Number conceived**	**Conception rate**	**Contribution towards conception**	**Chi-square value**	***P*-value**
**Breed type**								
Bonsmara	57	100	56	28	50.00	10.26	1.4204	0.4915
Brahman	30	100	30	19	63.33	6.96		
Nguni	184	100	177	95	53.67	34.80		
Other^‡^	11	100	10	09	90.00	3.29		
Total	282	100	273	151	55.31	55.31		
**Body frame size**								
Small	53	100	53	26	49.06	9.52	5.9008	0.0523
Medium	212	100	203	111	54.68	40.66		
Large	17	100	17	14	82.35	5.13		
Total	282	100	273	151	55.31	55.31		

‡ *Other = (Afrikaner = 2, Drakensberg = 3, Simmentaler = 1 and non-descript = 4)*.

**Table 3:** Effect of parity and age on synchronisation response and conception rate of cows under communal and emerging farming systems.

**Variables**	**Number synchronised**	**Synchronisation response rate**	**Number of cows inseminated**	**Number conceived**	**Conception rate**	**Contribution towards conception**	**Chi-square value**	***P*-value**
**Parity**								
First	101	100	98	55	56.12	20.15	3.1329	0.5358
Second	79	100	74	38	51.35	13.92		
Third	48	100	47	29	61.70	10.62		
Fourth	22	100	22	15	68.18	5.50		
Fifth+	07	100	07	05	71.43	1.83		
Unknown	25	100	25	09	36.00	3.29		
Total	282	100	273	151	55.31	55.31		
**Age (years)**								
4	107	100	104	60	57.96	21.98	9.6947	0.0844
5	51	100	49	24	48.97	8.79		
6	54	100	53	26	49.06	9.52		
7	27	100	24	17	70.83	6.23		
8+	18	100	18	15	83.33	5.50		
Unknown	25	100	25	09	36.00	3.29		
Total	282	100	273	151	55.31	55.31		

**Table 4:** Effect of body condition score and lactation status on synchronisation response and conception rate of cows under communal and emerging farming systems.

**Variables**	**Number synchronised**	**Synchronisation response rate**	**Number of cows inseminated**	**Number conceived**	**Conception rate**	**Contribution towards conception**	**Chi-square value**	***P*-value**
**Body condition score**								
≤2.5	68	100	65	43	66.15	15.75	10.1481	0.0063^*^
3	180	100	176	85	48.30	31.14		
≥3.5	34	100	32	23	71.88	8.42		
Total	282	100	273	151	55.31	55.31		
**Lactation status**								
Dry	179	100	172	98	56.98	35.90	0.8145	0.3668
Lactating	103	100	101	53	52.48	19.41		
Total	53	100	273	151	55.31	55.31		

* *Significant relationship (not independent) (P < 0.05)*.

### Calving and Survival Rate Following Timed Artificial Insemination

A calving and survival rate of 48 and 100%, respectively, was recorded in the current study (Table 5). Calving rate was not independent of province and district. The calving rate in MP (58%) and KZN (54%) was significantly higher than that obtained in LP (36%). There was no significant difference (*P* > 0.05) between the calving rate in MP and KZN provinces. In LP province, the Vhembe (44%) district had a significantly higher (*P* <0.05) calving rate than that recorded in Capricorn (32%), Mopani (23%), and the Waterberg (30%) districts. In Mpumalanga province, Gert Sibande (61%) had a significantly higher calving rate than the Ehlanzeni (50%) district. In KZN province, there was no significant difference between Zululand (50%) and Harry Gwala (61%) district. There was no significant difference (*P* > 0.05) between the Ehlanzeni district of Mpumalanga, Zululand, and Harry Gwala of KZN and the Vhembe district of LP.

**Table 5:** Effect of province and districts on calving and survival rate of cows under communal and emerging farming systems.

**Variables**	**Number of cows inseminated**	**Number of cows calved**	**Calving rate**	**Survival rate**	**Contribution towards calving**	**Chi-square value**	***P*-value**
**Province**								
Limpopo	108	39	36.11	100	14.29	43.2016	0.0001^*^
Mpumalanga	91	53	58.24	100	10.41		
KZN	74	40	54.05	100	14.65		
Total	273	132	48.35	100	48.35		
**Districts**								
Vhembe	48	21	43.73	100	7.69	15.3765	0.0001^*^
Capricorn	37	12	32.43	100	4.39		
Mopani	13	03	23.08	100	1.10		
Waterberg	10	03	30.00	100	1.10		
Gert Sibande	69	42	60.87	100	15.38		
Ehlanzeni	22	11	50.00	100	4.03		
Zululand	46	23	50.00	100	8.43		
Harry Gwala	28	17	60.71	100	6.23		
Total	273	132	48.35	100	48.35		

* *Significant relationship (not independent) (P < 0.05)*.

Breed type and body frame size had no association with calving and survival rate (Table 6). However, the Brahman (53%) breed type had a higher calving rate than Bonsmara (46%) and Nguni (48%) breed type cows. Again, there was a small group classed as other that were neglected in the further discussion because of the group's small size.

**Table 6:** Effect of breed and body frame size on calving and survival rate of cows under communal and emerging farming systems.

**Variables**	**Number of cows inseminated**	**Number of cows calved**	**Calving rate**	**Survival rate**	**Contribution towards calving**	**Chi-square value**	***P*-value**
**Breed type**								
Bonsmara	56	26	46.43	100	9.52	2.0089	0.3662
Brahman	30	16	53.33	100	5.86		
Nguni	177	84	47.46	100	30.77		
Other^‡^	10	06	60.00	100	2.20		
Total	273	132	48.35	100	48.35		
**Body frame size**								
Small	53	23	26	100	8.43	4.9067	0.0860
Medium	212	98	111	100	35.89		
Large	17	11	14	100	4.03		
Total	282	132	151	100	48.35		

‡ *Other = (Afrikaner = 2, Drakensberg = 3, Simmentaler = 1 and non-descript = 4)*.

Cows with a large body frame (65%) had the highest calving rate compared to small (43%) and medium (48%) framed cows. Parity and age had to association with calving and survival rate (Table 7). However, cows in the fifth+ (71%) parity had the highest calving rate than the first (46%) parity cow, which also happens to be the least calving recorded in the study. Additionally, cows aged 8+ (67%) had the highest calving, whereas the least calving was recorded in cows aged 6 (45%) years. Chi-Test of Independence showed that body condition score was not independent of calving (*P* < 0.05; Table 8). Cows of body condition score of ≤2.5 (60%) had a significantly higher (*P* < 0.05) calving rate than those with body condition score of 3 (43%). However, there was no significant difference (*P* > 0.05) between cows of body condition scores of 3 and ≥3.5. Lactation status of a cow had no significant relationship (*P* > 0.05) with calving rate under the low-income beef sector.

**Table 7:** Effect of parity and age on calving and survival rate of cows under communal and emerging farming systems.

**Variables**	**Number of cows inseminated**	**Number of cows calved**	**Calving rate**	**Survival rate**	**Contribution towards calving**	**Chi-square value**	***P*-value**
**Parity**								
First	98	45	45.92	100	16.48	1.5090	0.8251
Second	74	36	48.65	100	13.19		
Third	47	23	48.94	100	8.43		
Fourth	22	14	63.64	100	5.13		
Fifth+	07	05	71.43	100	1.83		
Unknown	25	09	36.00	100	3.29		
Total	273	132	48.35	100	48.35		
**Age (years)**								
4	104	104	48.08	100	21.98	2.0669	0.8398
5	49	49	48.98	100	8.79		
6	53	53	45.28	100	9.52		
7	24	24	54.17	100	6.23		
8+	18	18	66.67	100	5.50		
Unknown	25	25	36.00	100	3.29		
Total	273	273	48.35	100	55.31		

**Table 8:** Effect of body condition score and lactation status on calving and survival rate of cows under communal and emerging farming systems.

**Variables**	**Number of cows inseminated**	**Number of cows calved**	**Calving rate**	**Survival rate**	**Contribution towards calving**	**Chi-square value**	***P*-value**
**Body condition score**								
≤2.5	65	39	60.00	100	14.28	9.8982	0.0076^*^
3	176	76	43.18	100	27.84		
≥3.5	32	17	53.12	100	6.23		
Total	273	132	48.35	100	48.35		
**Lactation status**								
Dry	172	172	98	100	30.77	1.4151	0.2342
Lactating	101	101	53	100	17.58		
Total	273	273	151	100	48.35		

* *Significant relationship (not independent) (P < 0.05)*.

### Stakeholder Perception Analysis

Cattle farmers and Provincial Departments of Agriculture (PDAs) were key stakeholders in the low-income beef sector in South Africa. A total of 28 cattle stakeholders were identified for the interview. A multitude of challenges emerged that compromise the implementation and adoption of reproductive technologies under the low-income beef sector (Table 9). Challenges were grouped into three categories, namely; human interference, lack of resources and those emanating from natural causes.

**Table 9:** Challenges to the accessibility of reproductive technologies by low-income beef farmers in South Africa.

**Human interference**	**Lack of resources**	**Natural causes**
Expansion of dwelling areas	Inability to supplement animals	Sores and injuries due to horns and thorns
Dispute by stakeholders	Inadequate infrastructure	Drought and dry season
Stock theft	Lack of medication	Lightning
Delayed and lack of government serves	Lack of access to the market	High pre-weaning mortality
Inbreeding	Shortage of grazing land	Predators
Poor cattle management	Shortage of bulls	Global warning
Lack of cattle farming strategy	Lack of good breeding materials	High incidence of abortion
Fire outbreak	Insufficient government services	
Labour regulations		

The two most common constraints relating to human interference that was perceived to affect the implementation and adoption of reproductive technologies under the low-income beef sector as given by respondents in all provinces in order of rankings were: stock theft and expansion of dwelling areas. Two prominent challenges emanating from lack of resources were that potentially can reduce the accessibility of reproduction technologies in the low-income beef sector in order of ranking were: inadequate infrastructure and lack of access to the market. Perceived main challenges of natural causes in the three provinces that potentially reduces access to reproductive technologies were: drought and dry season, and diseases.

## Discussion

### Cow Performance

#### Oestrous Synchronisation Response

Results recorded in the current study are comparable with those reported by Martinez et al. (24) in beef cattle when EB and progesterone were used, and those by Maqhashu (25) who reported 99 and 100% synchronisation response rate in cows under low-income beef sector in LP and KZN, respectively. Rahman et al. (26) also reported a 100% synchronisation response rate in supplemented crossbred cows in Bangladesh. However, the results differ from those reported by other authors. Raphalalani (27) reported an overall synchronisation response rate of 75% in LP under the emerging farming sector. Cabara and Velicevici (28) reported a synchronisation response rate of 63 percent in smallholder dairy cows in Romania. According to Martinez et al. (24), the use of EB in place of Gonadotropin-Releasing Hormone (GnRH) improves the expression of oestrous in beef cattle. Tropical breeds have a particular temperament that results in a “silent” or “missed” heat (29). Furthermore, tropical breeds have smaller corpus luteum that affect serum progesterone levels and subsequently lower response to oestrogen and oestrus behaviour (29, 30). Bó et al. (29) and Alvarez et al. (30) suggested that exposure to stressors (environmental or animal derived) could decrease oestrus expression and normal ovulation. Alnimer et al. (31) and Hansen (32) described heat stress among the factors affecting the reproduction and oestrous manifestation in cows. Stress emanating from excessive heat can reduce follicular development, which in turn reduces oocyte growth, lowering the expression of oestrous behaviour and increasing undetected oestrous (28). The anticipation from this study was that it would be difficult to synchronise beef cattle under low-income sector since the project was conducted during the summer months (October to March) when the environmental temperature averages 30°C.

The heat mount detectors assisted in the detection of cows on heat. The detector requires a cow to stand immobile and be mounted by another animal in order for the patch to turn red. Mounting behaviour can affect the synchronisation response rate. The number of mounts increased as the number of cows in oestrous increased (33). Discrepancies in the use of heat mount detectors cannot be ruled out. For example, an animal may move away after another animal attempt to mount it under veld farming conditions. An animal could also use a tree branch for scratching itself, causing the detector to give a false positive reading.

#### Conception Rate of Synchronised Cows

Fertilisation failure is the most important factor reducing the accomplishment of reproductive programmes in dairy and beef cattle. The current study observed an overall conception rate of 55%. This result falls within the suggested conception rate of between 25 and 67% as reported by Baruselli et al. (34), conception rates of between 50 and 90% suggested by Borges et al. (35) and conception rates of between 40 and 60% suggested by Woldu et al. (36). The results show an improvement when compared with previous documented indigenous low-income beef cattle conception rates such as 27.06% in Ethiopia (37), 41% in South Africa (27), 47.5% in Ethiopia (38), 48.3% in Ethiopia (36), and 47% in South Africa (25). The results are lower than those stated by other researchers such as Ali et al. (38) who reported a conception rate of 62.5% in Ethiopia, Garcia et al. (39) who reported a conception rate of 61.4% in the Amazon basin of Peru, and Baruselli et al. (34) who reported a conception rate of 85% in Brazil.

The differences in conception might be due to different hormones and management systems. Studies by Desalegn (37), Mukasa-Mugerwa et al. (40), and Woldu et al. (36) relied on heat detection before insemination based on the “am-pm guideline.” In their studies, cows observed to be on heat in the morning were inseminated that very afternoon, and those identified in the afternoon were inseminated the following morning. The peak time for an animal to display oestrus is usually overnight, and its duration is short (34, 41, 42), it is possible to inseminate the animal late due to poor oestrus detection processes by the farmers. Furthermore, under extensive communal and emerging beef cattle farming, animals graze extensively in camps at walking distance from the homestead, which might also compromise the oestrous detection process. However, Chebel et al. (43) in California (USA) found that the type of insemination protocol, timed AI or AI upon oestrus detection, results in similar conception rates. Furthermore, studies by Baruselli et al. (34) and Raphalalani (27) used the ovsynch protocol similar to the one used in the current study with varying results, proving that conception is a multifaceted product. Stress emanating from excessive heat is a major cause of low cow fertility and pregnancy rate following AI (29). Infertility and subfertility of cows under communal and emerging systems may also have reduced the conception rate.

In the present study, the province had a significant effect on conception. The conception was highest in KZN (66%) rather than in MP (60%) and LP (44%). The vegetation and available grazing are dependent on rainfall, and KZN is South Africa's most watered province with an average of over 1,000 mm of rainfall per annum (15, 19). An abundance of vegetation in an area is manifested through good body conditions of the animals, which in turn affects the conception rate (26, 34, 41). Cows with a body condition score of ≥3.5 (72%) had the highest conception rate compared to those with a score of 3 (48%) and ≤ 2.5 (66%). Bó et al. (44) and Woldu et al. (36) observed an increase in conception rate with increased body condition score when working with village cows in Brazil and Ethiopia, respectively. The nutritional status of the animal affects ovarian function, which in turn affects their reproductive performance (45).

Cows that were not suckling a calf had a higher conception rate (57%) than those that were lactating (53%), although the difference was not significant (P > 0.05). Beef cattle are characterised by prolonged post-partum anoestrous, which will affect their conception rate (46). Poor management such as lack of weaning can add to this problem. When weaning is delayed, cows take much longer to restore their body condition, affecting their conception rate.

There was no difference in conception rate between animals of different sizes. However, under natural farming systems where feed availability is seasonal, farming with small to medium framed animals such as the Nguni can be beneficial. The Nguni has a low maintenance feed requirement and is adapted to local conditions (47). When compared with an equal amount of data, the Nguni has demonstrated that it can perform better than the Bonsmara and Brahman (27).

#### Calving and Survival Rate

Calving rate has been used to measure reproductive performance under the low-income beef sector in South Africa. The current study recorded an overall calving rate of 48%. A conception rate of 55% was recorded, thus giving a pregnancy loss of 7%. The calving results are an improvement on the 40% reported under natural mating in the low-income beef sector (8, 48). Pursley et al. (49) recorded an overall calving rate of 29% with a pregnancy loss of 20% in dairy herds in the US. Mokantla et al. (3) recorded a calving rate of 38% under natural service in village farming areas of South Africa with a pregnancy loss of 12%. The current pregnancy loss is rather lower than that reported by Pursley et al. (49) and Mokantla et al. (3).

The current study recorded a 100% survival rate. However, since beef cattle under the low-income sector graze on rangelands that are a distance at times of about 12 km from homesteads (45), farmers can have missed out on some of the calves that might have died immediately after calving. Cattle are hardly kraaled in many villages unless they are to be worked on. Leaving cattle out in bushy dense veld can potentially expose the newly born and the young to predators. Some villages are located close to wildlife reserves (50), and the potential for wildlife such as hyenas, wild dogs, and leopards to scavenge on the young and weak is high.

Though parity was used as a qualifying criterion during selection, not all cows inseminated conceived. Cows that never conceive following the service may be pointing to some degree of infertility and subfertility in the herd (3). Reproductive diseases are amongst the many factors affecting conception, pregnancy rate and calving rate (45, 51). Again, the calving rate recorded in this study could have been higher had it not been due to the drought that South Africa experienced between 2015 and 2016. According to Munyai (10), drought in 1 year results in lower calving the following year. Therefore, drought conditions may have been the cause of pregnancy losses and a lower calving rate.

The body weight and body condition score affects the reproductive performance of the animal and are directly associated with the nutritional status of an animal (46, 51, 52). Calving rate increases with an improved body condition score (27, 36, 44). However, in the current study, cows with a body condition score of ≤ 2.5 had a significantly higher calving rate than those with a body condition score of 3 and ≥3.5. These results might have been influenced by human error on condition score judgement during data collection. Three different enumerators, though trained on body condition scoring (1–5, 1 = thin, 5 = obese), worked independently in different provinces. However, Bó and Baruselli et al. (34) reported that cows must have a BCS higher than 2.5 and ideally 3 to achieve a pregnancy rate of 50% or more. However, the same authors indicated that equine chorionic gonadotropin (eCG) administration during synchronisation allows for a pregnancy rate of close to 50% in cows with a BCS of ≤2.5. In the current study, an ovsynch protocol that uses progesterone and EB was used instead of eCG.

### Perception Analysis Study

#### Human Interference

Crime statistics reports and surveys in South Africa indicate that rural livestock farmers are affected mainly by stock theft (53). Hangara (54) and Malekano (55) reported about stock theft as a challenge facing communal cattle farmers in Namibia and Malawi, respectively. Stock theft was not as rife during apartheid years in South Africa as it is now. Poverty and unemployment are postulated as the prime cause of theft in these historical settings. Nengovhela (16) reported that most farmers feel that there was better maintenance of the rule of law during apartheid than it is now. Geldenhuys (56) explains that stock theft is an ever-increasing, unsettling and destructive reality affecting all sectors of the farming community.

According to the South African Police Service (57), stock theft takes place more often than other forms of criminality, and it is a much more serious threat in South African regions bordering other countries, such as the Eastern Cape, Mpumalanga, Free State, KwaZulu-Natal, North West, and Limpopo. Ever since the fall of apartheid, there has been a rapid expansion of villages and dwellings. Many small, medium, and large enterprises, and tourist attraction businesses such as overnight accommodation and holiday resorts have also been rising. These mushrooming businesses are all at the expense of land originally allocated for grazing and field crops. Nengovhela (16) also reported the expansion of the dwellings at the expense of available grazing as a challenge facing cattle farmers in the low-income sector.

#### Lack of Resources

Most low-income beef cattle farmers are located in remote rural areas; and lack the capital to fund the development of infrastructure and construction of handling facilities (58). According to Nkosi and Kirsten (59), inadequate infrastructure merely takes away the limited incentives available to rural farmers. Gwala (12) reported that the poor state of access roads and lack of transport facilities in rural Eastern Cape Province. Almost all the cattle infrastructure currently in existence there was built by the Department of Agriculture (6, 12). According to Frisch (58), in communities with facilities, are either in a poor state or non-functional because they were erected some 50–60 years ago, and farmers do not have the cash to maintain them. The lack of infrastructure can seriously hinder development initiatives such as the implementation of reproductive technologies in rural farming communities. Ruijs et al. (60) argued that investment in infrastructure has an important positive effect on development. Most of the handling facilities in the hands of communal cattle farmers in Limpopo, Mpumalanga, and KwaZulu-Natal provinces were dilapidated since repairs rarely happen.

According to Bekure and Tilahun (61), marketing provides the mechanism whereby producers transact their livestock for notes. Approximately 40% of livestock in South Africa is in the hands of low-income farmers (62). The transition from communal and emerging sectors towards commercial production is dependent on market access (63, 64). Bailey et al. (65), Montshwe (66) and Musenwa et al. (67) identified poor market infrastructure, increasing market price variability, high transaction costs, and low purchasing power of buyers as major problems resulting in limited market participation. Ainslie et al. (7) identified cultural values and poor production practises rather than market failures as significant causes of limited market participation.

#### Natural Causes

According to Mathieu and Yves (68), drought and dry seasons are regular and recurrent features of the South African climate. Their impact on society depends on their intensity and durations. Though this challenge is recurrent, low-income beef cattle farmers had no strategy to deal with the challenge. The results of drought are severe water and feed shortages, and the death of animals. These, in turn, will affect the body condition score and the ability of animals to cycle and conceive, negatively impacting the implementation of reproductive technologies. South Africa experienced severe drought in 2015 through to 2016, and a large number of cattle died during this period. Motiang and Webb (69) found that farmers do not dispose-of their animals even when there is an anticipation of drought. In South Africa, feed availability follows the rainfall patterns with more grazing in summer than in winter. The winter season (May to July) is dry with no rainfall and is characterised by dry grasses of low quality.

Parasites and diseases are among the major constraints faced by communal and emerging beef cattle farmers in developing countries due to several reasons including the unavailability and high cost of drugs and medicines (70). The most problematic diseases listed by respondents were tick-borne diseases such as heart water and redwater, lumpy skin disease, FMD, and abscesses. This scenario is comparable with many authors (71–73) who reported tick-borne diseases as the leading cause of substantial losses in cattle production, reduced productivity, the decline in fertility, and often death. However, cattle farmers were aware that indigenous cattle breeds such as the Ngunis are tick and disease resistant, and that they should be promoted since they will fit the low-input low-output production system common in rural setups.

### Conclusion

The results of this study have demonstrated that village cows, irrespective of the province, breed type, parity level, age, body condition score, frame size, lactation status, and geographic location can be successfully synchronised and artificially inseminated with frozen-thawed semen and conceive. Calving rates recorded during the current study were higher than those recorded under natural mating. There was an increase in conception rate with an increase in body condition score. So, good nutrition is essential for an improved conception rate. Cows that were lactating during the implementation of the assisted reproductive technologies project had more chances to calve than those that were not lactating, thus affirming that calving is a good measure of the reproductive efficiency in a herd. Large framed animals had a higher conception rate than small and medium-framed animals. However, their feed maintenance requirement will compromise their performance in lower rainfall areas such as the Limpopo Province. It is advisable for farmers in lower rainfall areas to farm with small to medium framed animals such as the Ngunis with lower feed maintenance requirements. There are many of challenges that can reduce the accessibility of reproductive technologies under the low-income beef sector in South Africa. Low-income beef cattle farmers need to identify challenges within their reach instead of waiting for the government. The government is stretched with ever-rising social challenges and depleting resources. All stakeholders that participated in the project agreed that the observed results were encouraging and serves as an inspiration for the adoption of the practise. Other provinces in the country that were not participating in the study closely monitored the practical outcome of the project. It did not come as a surprise that KwaZulu-Natal that participated in the study, and North West Province that did not participate in the project, adopted ART practises to support reproductive performance in the low-income beef sector.

## Data Availability Statement

The raw data supporting the conclusions of this article will be made available by the authors, without undue reservation.

## Ethics Statement

The animal study was reviewed and approved by University of Fort Hare Animal Research Ethics (NED011SMUG01). Written informed consent was obtained from the owners for the participation of their animals in this study.

## Author Contributions

TM, TN, and KN: conceptualisation, methodology, and validation. TN: funding acquisition. TM and TN: organisation and data collection. TM and KN: data screening and analysis. TM and NN: administration and writing. TN and KN: reviewing and editing. All authors had read and agreed to the published version of the manuscript.

## Conflict of Interest

The authors declare that the research was conducted in the absence of any commercial or financial relationships that could be construed as a potential conflict of interest.

## Publisher's Note

All claims expressed in this article are solely those of the authors and do not necessarily represent those of their affiliated organizations, or those of the publisher, the editors and the reviewers. Any product that may be evaluated in this article, or claim that may be made by its manufacturer, is not guaranteed or endorsed by the publisher.
